# NLRP3 Inflammasome Activation Is Involved in LPA_1_-Mediated Brain Injury after Transient Focal Cerebral Ischemia

**DOI:** 10.3390/ijms21228595

**Published:** 2020-11-14

**Authors:** Chi-Ho Lee, Arjun Sapkota, Bhakta Prasad Gaire, Ji Woong Choi

**Affiliations:** Laboratory of Neuropharmacology, College of Pharmacy and Gachon Institute of Pharmaceutical Sciences, Gachon University, Incheon 21936, Korea; lch7835@nate.com (C.-H.L.); sapkotaa07@gmail.com (A.S.); samarpanbp@gmail.com (B.P.G.)

**Keywords:** LPA_1_, transient middle cerebral artery occlusion, NLRP3 inflammasome, bone marrow-derived macrophage, LPA, lipopolysaccharide

## Abstract

Lysophosphatidic acid receptor 1 (LPA_1_) contributes to brain injury following transient focal cerebral ischemia. However, the mechanism remains unclear. Here, we investigated whether nucleotide-binding oligomerization domain-like receptor family pyrin domain containing 3 (NLRP3) inflammasome activation might be an underlying mechanism involved in the pathogenesis of brain injury associated with LPA_1_ following ischemic challenge with transient middle cerebral artery occlusion (tMCAO). Suppressing LPA_1_ activity by its antagonist attenuated NLRP3 upregulation in the penumbra and ischemic core regions, particularly in ionized calcium-binding adapter molecule 1 (Iba1)-expressing cells like macrophages of mouse after tMCAO challenge. It also suppressed NLRP3 inflammasome activation, such as caspase-1 activation, interleukin 1β (IL-1β) maturation, and apoptosis-associated speck-like protein containing a caspase recruitment domain (ASC) speck formation, in a post-ischemic brain. The role of LPA_1_ in NLRP3 inflammasome activation was confirmed in vitro using lipopolysaccharide-primed bone marrow-derived macrophages, followed by LPA exposure. Suppressing LPA_1_ activity by either pharmacological antagonism or genetic knockdown attenuated NLRP3 upregulation, caspase-1 activation, IL-1β maturation, and IL-1β secretion in these cells. Furthermore, nuclear factor-κB (NF-κB), extracellular signal-regulated kinase 1/2 (ERK1/2), and p38 were found to be LPA_1_-dependent effector pathways in these cells. Collectively, results of the current study first demonstrate that LPA_1_ could contribute to ischemic brain injury by activating NLRP3 inflammasome with underlying effector mechanisms.

## 1. Introduction

Lysophosphatidic acid (LPA) is a bioactive lysophospholipid that possesses diverse physiological and pathological functions throughout the body by activating its specific six G protein-coupled receptors (LPA_1–6_) [1,2]. LPA receptors have been of considerable therapeutic interest for drug development to treat many diseases [2,3]. In particular, targeting LPA_1_ has become a promising strategy for drug development due to clinical trials for pulmonary fibrosis (ClinicalTrials.gov ID: NCT01766817) and psoriasis (ClinicalTrials.gov ID: NCT02763969). In case of cerebral ischemia that occurs by a sudden blockade of blood supply in the brain and causes severe brain damage, LPA_1_ has been identified as a pathogenic factor for brain injury after ischemic challenge. Suppressing LPA_1_ activity by either pharmacological antagonism or genetic deletion can reduce brain damage, such as brain infarction, functional neurological deficits, and pain [4,5,6]. In particular, administration of an LPA_1_ antagonist (AM095) immediately after reperfusion significantly reduced brain infarction and neurological deficit score in mice at 1 day and 3 days after transient middle cerebral artery occlusion (tMCAO) challenge [4], supporting that LPA_1_ could contribute to brain injuries after ischemic challenge. As an underlying pathogenesis, LPA_1_ can regulate immune responses in post-ischemic brain by upregulating proinflammatory cytokines and increasing numbers of cells expressing ionized calcium-binding adapter molecule 1 (Iba1), a marker for activated microglia or infiltrated macrophages [4]. However, how LPA_1_ can contribute to brain injuries following ischemic challenge remains unclear.

Nucleotide-binding oligomerization domain-like receptor family pyrin domain containing 3 (NLRP3) is a sensor for various pathogen- and host-derived factors [7]. Upon activation, it forms a complex called NLRP3 inflammasome, leading to the production of proinflammatory cytokines, interleukin 1β (IL-1β) and IL-18 [8,9]. NLRP3 inflammasome activation contributes to tissue injuries in various diseases throughout the body [10,11,12,13,14]. In cerebral ischemia, the importance of NLRP3 inflammasome activation as a pathogenic mediator has been suggested [15]. NLRP3 is upregulated and NLRP3 inflammasome is activated in post-ischemic brain [15]. A genetic deletion of NLRP3 can decrease brain damage in mice after ischemic challenge [15]. Its pharmacological suppression can ameliorate ischemic injury and neurovascular complications in cellular and animal models of cerebral ischemia [16,17]. Recently, it was suggested that LPA can regulate NLRP3 inflammasome activation in lipopolysaccharide (LPS)-primed bone marrow-derived macrophages (BMDMs) [18]. Considering increased amounts of LPA in human ischemic patients [19] and animal models of cerebral ischemia [5,20], LPA signaling may regulate NLRP3 inflammasome activation in injured brain following ischemic challenge. Moreover, LPA_1_ may be responsible for NLRP3 inflammasome activation.

To test such possible role of LPA_1_, the current study determined whether suppressing LPA_1_ activity by its specific antagonist could ameliorate NLRP3 upregulation in an injured brain of mouse with tMCAO challenge through biochemical and immunohistochemical analyses. We also determined whether it could suppress NLRP3 inflammasome activation by measuring caspase-1 activation and IL-1β maturation in a post-ischemic brain. Through the previously reported in vitro system using LPS-primed BMDMs, followed by an exposure to LPA [18], we confirmed the role of LPA_1_ in NLRP3 inflammasome activation. Furthermore, we determined which LPA_1_-dependent pathways were involved in NLRP3 inflammasome activation.

## 2. Results

### 2.1. Suppressing LPA_1_ Activity Attenuates NLRP3 Inflammasome Activation in an Injured Brain after tMCAO Challenge

**Figure 1:**
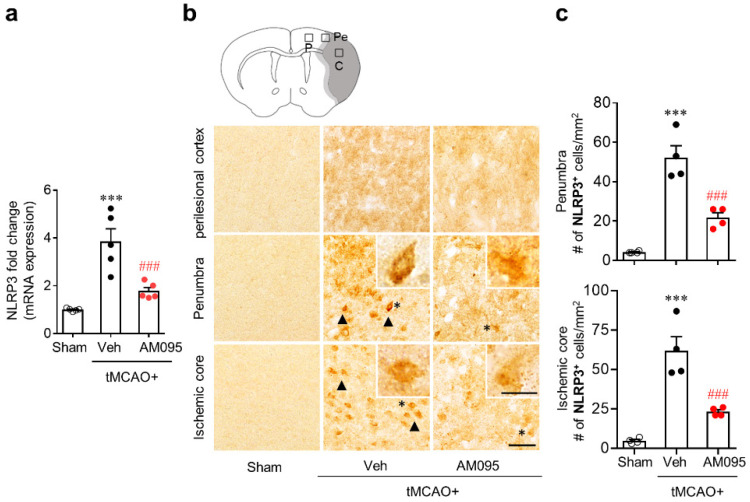
Lysophosphatidic acid receptor 1 (LPA_1_) antagonist attenuates nucleotide-binding oligomerization domain-like receptor family pyrin domain containing 3 (NLRP3) upregulation in injured brains after transient middle cerebral artery occlusion (tMCAO) challenge. Mice were induced with tMCAO. AM095 (30 mg/kg, p.o.) was administered immediately after reperfusion. (**a**) mRNA expression levels of NLRP3 at one day after ischemic challenge by qPCR analysis. (**b**,**c**) NLRP3 expression determined by immunohistochemistry at one day after tMCAO challenge. (**b)** Representative images of NLRP3-immunopositive cells in perilesional cortex (P), penumbra (Pe), and ischemic core (C) regions. A graphical abstract image at the top shows cerebral areas where images in top, middle, and bottom planes are taken. Arrows indicate NLRP3-immunopostive cells. Cells in the insets are indicated as asterisks (*). (**c**) Quantification of NLRP3-immunopositive cells in penumbra and ischemic core regions. Scale bar, 50 µm (inset, 10 µm). *n* = 4 mice per group. *** *p* < 0.001 versus sham. ^###^ *p* < 0.001 versus vehicle-administered tMCAO group. Neurological deficit scores of mice used for qPCR analysis and immunohistochemistry are presented as a supplementary figure (Figure S1a,b).

To address whether LPA_1_ could regulate NLRP3 expression in an injured brain after tMCAO challenge, we first determined mRNA expression levels of NLRP3 by qPCR analysis. Results showed that mRNA expression levels of NLRP3 were significantly increased in the brain at one day after tMCAO challenge compared to those in the sham group (Figure 1a). This upregulation of NLRP3 mRNA in the injured brain was markedly attenuated by AM095 administration immediately after tMCAO challenge (Figure 1a). We further determined whether LPA_1_ could influence protein expression levels of NLRP3 in an injured brain after tMCAO challenge by immunohistochemical analysis. The number of NLRP3-immunopositive cells was significantly increased after tMCAO challenge (Figure 1b,c). This increase was observed mainly in the penumbra and ischemic core regions, but not in perilesional cortex (Figure 1b,c). AM095 administration significantly reduced the number of NLRP3-immunopositive cells in both the penumbra and ischemic core regions (Figure 1b,c).

**Figure 2:**
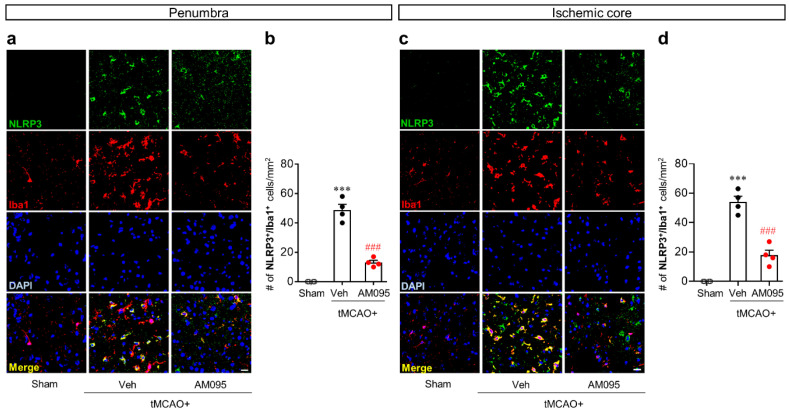
LPA_1_ antagonist attenuates NLRP3 upregulation in ionized calcium-binding adapter molecule 1 (Iba1)-immunopostive cells of injured brains after tMCAO challenge. Mice were induced with tMCAO. AM095 (30 mg/kg) was administered immediately after reperfusion. NLRP3 expression in Iba1-immunopositive cells was determined in the penumbra and ischemic core regions at one day after tMCAO challenge by NLRP3/Iba1 double immunofluorescence. Representative image of NLRP3/Iba1-double immunopositive cells in the penumbra (**a**) and ischemic core regions (**c**) and quantification of their numbers (**b**,**d**) are shown. Scale bars, 20 µm. *n* = 4 mice per group. *** *p* < 0.001 versus sham. ^###^ *p* < 0.001 versus vehicle-administered tMCAO group.

In a post-ischemic brain, NLRP3 upregulation can occur in Iba1-immunopositive cells [15]. Its attenuation by AM095 administration was clearly observed in the penumbra and ischemic core regions (Figure 1b,c). Therefore, we further determined whether the observed attenuation could occur in Iba1-expressing cells of the penumbra and ischemic core regions by NLRP3/Iba1 double immunofluorescence. The number of NLRP3/Iba1-double immunopositive cells was significantly increased in both the penumbra (Figure 2a,b) and ischemic core regions (Figure 2c,d) after tMCAO challenge. AM095 administration significantly reduced the number of NLRP3/Iba1-double immunopositive cells in both regions (Figure 2).

**Figure 3:**
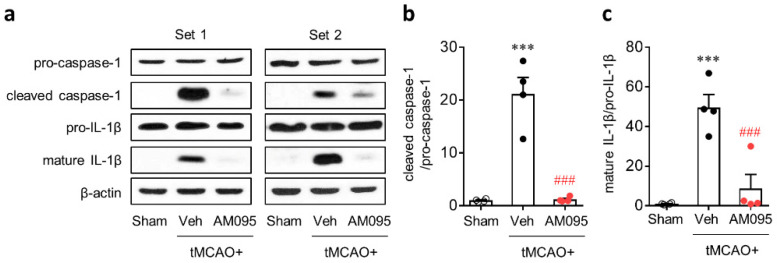
LPA_1_ antagonist attenuates NLRP3 inflammasome activation in injured brains after tMCAO challenge. Mice were induced with tMCAO. AM095 (30 mg/kg) was administered immediately after reperfusion. Caspase-1 activation and interleukin 1β (IL-1β) maturation were determined by Western blot analysis at 1 day after tMCAO challenge. Representative Western blots of pro-caspase-1, cleaved caspase-1, pro-IL-1β, and mature IL-1β (**a**) and quantification of caspase-1 activation (**b**) and IL-1β maturation (**c**) are shown. *n* = 4 mice per group. *** *p* < 0.001 versus sham. ^###^, *p* < 0.001 versus vehicle-administered tMCAO group. Neurological deficit scores of mice used for Western blot analysis are presented as a supplementary figure (Figure S1c).

To address whether LPA_1_ could regulate NLRP3 inflammasome activation in an injured brain after tMCAO challenge, caspase-1 activation and IL-1β maturation were determined by Western blot analysis. Caspase-1 activation and IL-1β maturation were known to be important events for the activation of NLRP3 inflammasome [21,22,23]. Expression levels of cleaved caspase-1 and mature IL-1β were significantly increased in the injured brain at one day after tMCAO challenge compared to those in the sham group (Figure 3). AM095 administration immediately after reperfusion markedly attenuated caspase-1 activation and IL-1β maturation (Figure 3).

**Figure 4:**
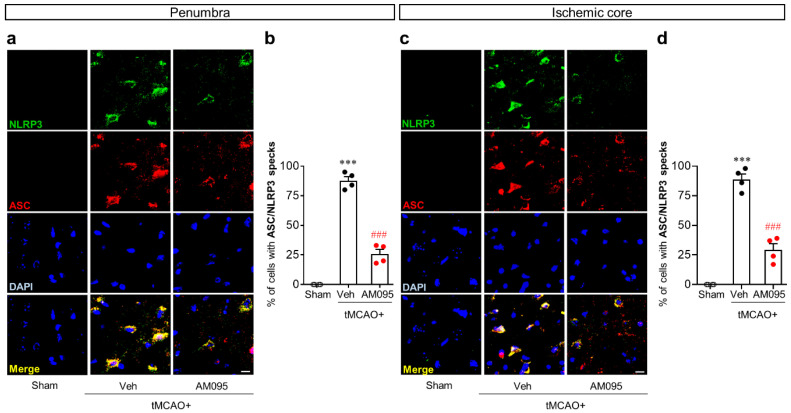
LPA_1_ antagonist attenuates apoptosis-associated speck-like protein containing a caspase recruitment domain (ASC) speck formation in injured brains after tMCAO challenge. Mice were induced with tMCAO. AM095 (30 mg/kg) was administered immediately after reperfusion. ASC speck formation in NLRP3-immunopositive cells was determined in the penumbra and ischemic core regions at one day after tMCAO challenge by ASC/NLRP3 double immunofluorescence. Representative images of ASC/NLRP3 speck formation in the penumbra (**a**) and ischemic core regions (**c**) and quantification of their numbers in percentage (**b**,**d**) are shown. Scale bars, 10 µm. *n* = 4 mice per group. *** *p* < 0.001 versus sham. ^###^, *p* < 0.001 versus vehicle-administered tMCAO group.

To further address the role of LPA_1_ in NLRP3 inflammasome activation in an injured brain after tMCAO challenge, the adaptor molecule apoptosis-associated speck-like protein containing a caspase recruitment domain (ASC) speck formation was determined by ASC/NLRP3 double immunofluorescence. The number of cells with ASC/NLRP3 specks was significantly increased in both the penumbra (Figure 4a,b) and ischemic core regions (Figure 4c,d) after tMCAO challenge. AM095 administration significantly reduced the number of cells with ASC/NLRP3 specks in both regions (Figure 4).

### 2.2. LPA_1_ Contributes to NLRP3 Upregulation and NLRP3 Inflammasome Activation in LPS-Primed Macrophages Followed by LPA Exposure

**Figure 5:**
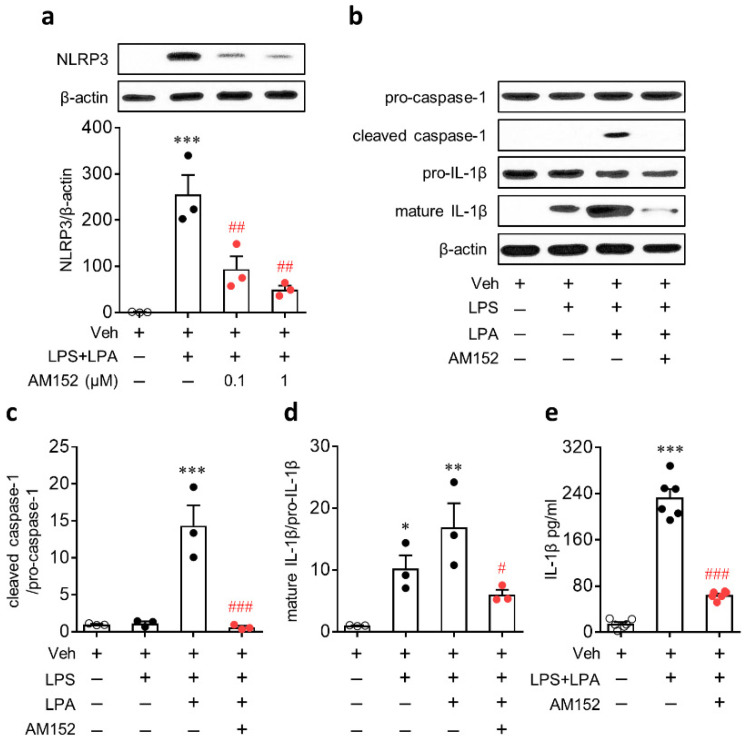
LPA_1_ antagonist attenuates LPA-induced NLRP3 inflammasome activation in lipopolysaccharide (LPS)-primed bone marrow-derived macrophages (BMDMs). Cells were primed with LPS (500 ng/mL) for 4 h, followed by an exposure to LPA (1 μM) for 1 h. (**a**–**d**) Protein expression levels of NLRP3, pro-caspase-1, cleaved caspase-1, pro-IL-1β, and mature IL-1β determined by Western blot analysis. (**a**) NLRP3 expression determined by Western blot analysis. AM152 was treated at different concentration (0; 0.1 and 1 μM). *n* = 3 per group. *** *p* < 0.001 versus control BMDMs (Veh). ^##^, *p* < 0.01 versus stimulated BMDMs (LPS-primed BMDMs, followed by an exposure to LPA; LPS+LPA). (**b**–**d**) Caspase-1 activation and IL-1β maturation determined by Western blot analysis. AM152 was treated at 1 μM. Representative Western blots of pro-caspase-1, cleaved caspase-1, pro-IL-1β, and mature IL-1β (**b**) and quantification of caspase-1 activation (**c**) and IL-1β maturation (**d**) are shown. *n* = 3 per group. * *p* < 0.05, ** *p* < 0.01, and *** *p* < 0.001 versus control BMDMs (Veh). ^#^ *p* < 0.05 and ^###^ *p* < 0.001 versus stimulated BMDMs (LPS-primed BMDMs, followed by an exposure to LPA; LPS+LPA). (**e**) Amounts of secreted IL-1β into the culture medium were measured by enzyme-linked immunosorbent assay (ELISA) analysis. *n* = 6 per group. *** *p* < 0.001 versus control BMDMs (Veh). ^###^ *p* < 0.001 versus stimulated BMDMs (LPS + LPA).

A previous study [15] and the current study (Figure 2) showed that NLRP3 was upregulated in cells expressing Iba1, a marker of infiltrated macrophages or activated microglia in post-ischemic brains [24,25]. These independent studies suggest that NLRP3 upregulation after ischemic challenge can occur in macrophages. Therefore, we studied the role of LPA_1_ in NLRP3 inflammasome activation in vitro using cultured BMDMs. To induce NLRP3 inflammasome activation, cells were primed with LPS and then exposed to LPA as previously reported [18] because ischemic challenge can increase amounts of LPA [5,20]. However, LPA itself cannot induce NLRP3 upregulation in BMDMs [18]. Instead, LPA was proven to enhance NLRP3 upregulation in LPS-primed BMDMs in a dose-dependent manner [18]. Therefore, we employed the same in vitro system of our previous study [18] to address the role of LPA_1_ in NLRP3 upregulation and NLRP3 inflammasome activation in macrophages. We first determined whether LPA_1_ could be involved in NLRP3 upregulation in macrophages. BMDMs were primed with LPS (500 ng/mL) for 4 h, followed by LPA exposure (1 µM) for 1 h. To determine the role of LPA_1_, cells were pretreated with AM152 (1 µM) for 30 min prior to LPS stimulation. As previously reported [18], NLRP3 was markedly upregulated in LPS-primed BMDMs, followed by LPA exposure (Figure 5a). However, AM152 significantly reduced expression levels of NLRP3 in LPS-primed BMDMs, followed by LPA exposure (Figure 5a).

Next, we determined whether LPA_1_ could regulate LPA-mediated NLRP3 inflammasome activation in LPS-primed BMDMs by analyzing caspase-1 activation and IL-1β production. LPA induced the caspase-1 activation and IL-1β maturation in LPS-primed BMDMs (Figure 5b–d). LPS alone did not induce caspase-1 activation in BMDMs (Figure 5b,c). However, LPS alone induced IL-1β maturation in BMDMs, but to a lesser extent than that in LPS-primed BMDMs, followed by LPA exposure (Figure 5b,c). IL-1β secretion into the culture medium was also elevated in LPS-primed BMDMs, followed by LPA exposure (Figure 5e). AM152 treatment significantly attenuated caspase-1 activation, IL-1β maturation, and IL-1β secretion (Figure 5b–e).

**Figure 6:**
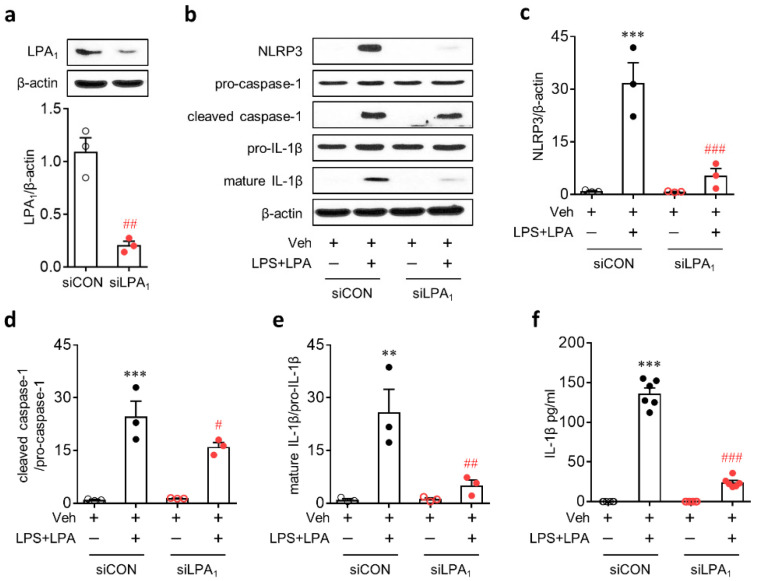
LPA_1_ knockdown attenuates LPA-induced NLRP3 inflammasome activation in LPS-primed BMDMs. Transfected BMDMs with non-target control small interfering RNA (siRNA) (siCON) or LPA_1_-specific siRNA (siLPA_1_) were primed with LPS (500 ng/mL) for 4 h, followed by an exposure to LPA (1 µM) for 1 h. (**a**) Knockdown efficiency of LPA_1_ siRNA determined by Western blot analysis. Representative Western blots of LPA_1_ and quantification. *n* = 3 per group. ^##^, *p* < 0.01 versus control siRNA (siCON)-transfected BMDMs. (**b–e**) Protein expression levels of NLRP3, pro-caspase-1, cleaved caspase-1, pro-IL-1β, and mature IL-1β determined by Western blot analysis. (**b**) Representative Western blots of NLRP3, pro-caspase-1, cleaved caspase-1, pro-IL-1β, and mature IL-1β. Quantification of NLRP3 upregulation (**c**), caspase-1 activation (**d**), and IL-1β maturation (**e**). *n* = 3 per group. ** *p* < 0.01 and *** *p* < 0.001 versus control BMDMs transfected with control siRNA (siCON + Veh). ^#^ *p* < 0.05, ^##^ *p* < 0.01, and ^###^ *p* < 0.001 versus stimulated BMDMs transfected with control siRNA (siCON + LPS + LPA). (**f**) Amounts of IL-1β secreted into the culture medium were measured by ELISA analysis. *n* = 6 per group. *** *p* < 0.001 versus control BMDMs transfected with control siRNA (siCON + Veh). ^###^ *p* < 0.001 versus stimulated BMDMs transfected with control siRNA (siCON + LPS + LPA).

We confirmed the role of LPA_1_ in NLRP3 inflammasome activation by a genetic knockdown using a specific small interfering RNA (siRNA) for LPA_1_. LPA_1_ knockdown (Figure 6a) significantly attenuated NLRP3 upregulation in LPS-primed BMDMs, followed by LPA exposure (Figure 6b,c). It also significantly attenuated NLRP3 inflammasome activation in these cells as evidenced by attenuated caspase-1 activation (Figure 6b,d), IL-1β maturation (Figure 6b,e), and IL-1β secretion (Figure 6f).

### 2.3. LPA_1_ Regulates NLRP3 Upregulation in LPS-Primed Macrophages Followed by LPA Exposure by Enhancing Nuclear Factor-κB (NF-κB) Translocation and Activating Extracellular Signal-Regulated Kinase 1/2 (ERK1/2) and p38 Mitogen-Activated Protein Kinase (MAPK)

**Figure 7:**
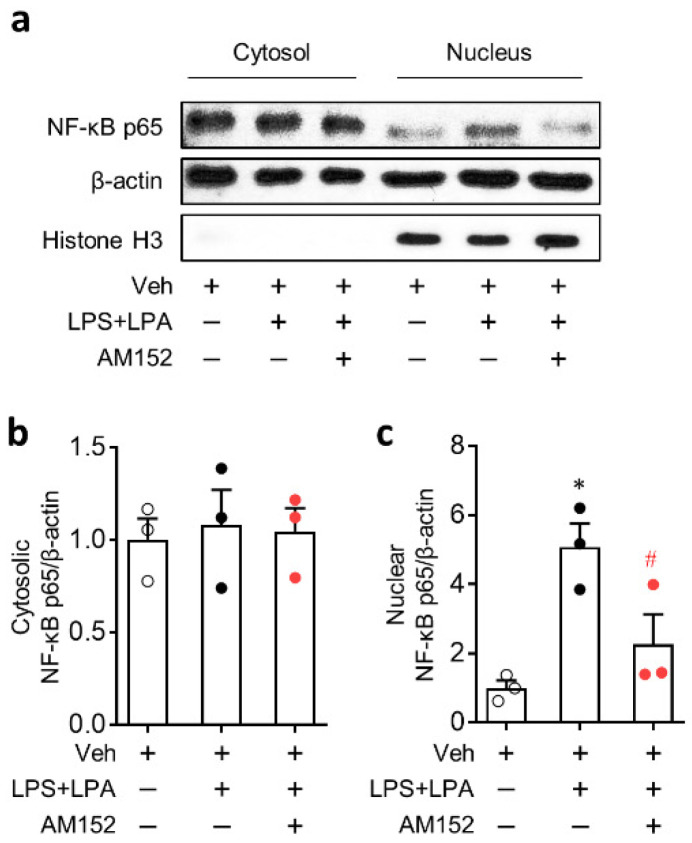
LPA_1_ antagonist inhibits LPA-induced nuclear factor-κB (NF-κB) activation in LPS-primed BMDMs. Cells were primed with LPS (500 ng/mL) for 4 h, followed by an exposure to LPA (1 μM) for 1 h. NF-κB activation was determined by comparing expression levels of cytosolic and nuclear NF-κB p65 based on Western blot analysis. Representative Western blots of cytosolic and nuclear NF-κB p65 (**a**) and quantification of NF-κB p65 translocation into the nucleus (**b,c**) are shown. *n* = 3 per group. * *p* < 0.05 versus control BMDMs (Veh). ^#^ *p* < 0.05 versus stimulated BMDMs (LPS + LPA).

NF-κB pathway is known to play an important role in NLRP3 inflammasome as a priming signal to induce NLRP3 upregulation [26,27,28]. Therefore, the role of LPA_1_ in NF-κB activation was determined by analyzing NF-κB translocation from the cytosol into the nucleus. LPA caused a marked translocation of NF-κB into the nucleus in LPS-primed BMDMs without affecting expression levels of NF-κB in the cytosol (Figure 7). When LPA_1_ activity was suppressed by AM152, such marked translocation was significantly weakened (Figure 7). We further determined that LPA_1_ could regulate NF-κB phosphorylation in BMDMs by Western blot analysis. NF-κB was markedly phosphorylated in LPS-primed BMDMs, followed by LPA exposure (Figure S2). This phosphorylation seemed to be induced solely by LPS priming because LPS itself also induced NF-κB phosphorylation to the similar extent (Figure S2). AM152 did not attenuate NF-κB phosphorylation in both LPS-treated BMDMs and LPS-primed BMDMs, followed by LPA exposure (Figure S2). These data indicate that LPA_1_ may activate NF-κB pathway by enhancing NF-κB translocation rather than its phosphorylation in LPS-primed BMDMs, followed by LPA exposure.

**Figure 8:**
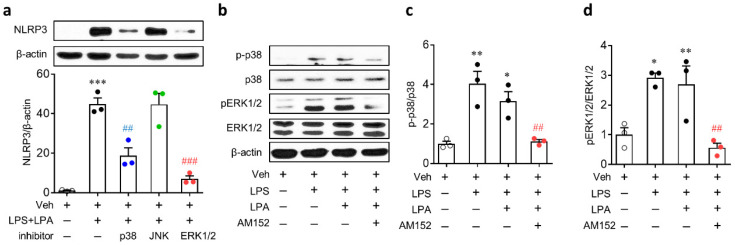
Activation of extracellular signal-regulated kinase 1/2 (ERK1/2) and p38 mitogen-activated protein kinase (MAPK) is involved in LPA_1_-mediated NLRP3 upregulation in LPS-primed BMDMs, followed by LPA exposure. Cells were primed with LPS (500 ng/mL) for 4 h, followed by an exposure to LPA (1 µM) for 1 h. Protein expression levels of NLRP3 and phosphorylation of p38 and ERK1/2 were determined by Western blots. (**a**) Effects of MAPK inhibitors on NLRP3 upregulation in LPS-primed BMDMs based on Western blot analysis. Representative blots and quantification data are shown. Cells were treated with each inhibitor for 30 min prior to LPS priming. *n* = 3 per group. *** *p* < 0.001 versus control BMDMs (Veh). ^##^ *p* < 0.01 and *^###^ p* < 0.001 versus stimulated BMDMs (LPS + LPA). (**b**-**d**) Effects of LPA_1_ antagonist on activation of p38 or ERK1/2 in LPS-primed BMDMs determined by Western blot analysis. Representative Western blots of phosphorylated p38 (p-p38), total p38 (p38), phosphorylated ERK1/2 (pERK1/2), total ERK1/2 (ERK1/2), and β-actin (**b**) and quantification of activation of p38 (**c**) and ERK1/2 (**d**) are shown. *n* = 3 per group. * *p* < 0.05 and ** *p* < 0.01 versus control BMDMs (Veh). ^##^ *p* < 0.01 versus stimulated BMDMs (LPS + LPA).

MAPK pathways, such as ERK1/2, p38, and c-Jun N-terminal kinase (JNK), are known to participate in NLRP3 inflammasome by upregulating NLRP3 [29,30,31]. These pathways are effector ones of LPA_1_ [32]. Therefore, the LPA/LPA_1_ signaling axis might regulate NLRP3 upregulation by activating MAPKs. To determine which MAPKs might be involved in NLRP3 upregulation in LPS-primed BMDMs, followed by LPA exposure, cells were treated with inhibitors of MAPKs, respectively, for 30 min prior to LPS priming. The upregulation of NLRP3 was significantly attenuated after inhibition of p38 and ERK1/2 but not after inhibition of JNK (Figure 8a). Whether ERK1/2 and p38 pathways could be regulated by LPA_1_ in LPS-primed BMDMs, followed by LPA exposure, was then determined. Both ERK1/2 and p38 were activated in LPS-primed BMDMs, followed by LPA exposure (Figure 8b–d). Similarly, they were also activated in cells treated with LPS alone (Figure 8b–d). However, AM152 significantly suppressed the activation of ERK1/2 and p38 in LPS-primed BMDMs, followed by LPA exposure (Figure 8b–d).

## 3. Discussion

**Figure 9:**
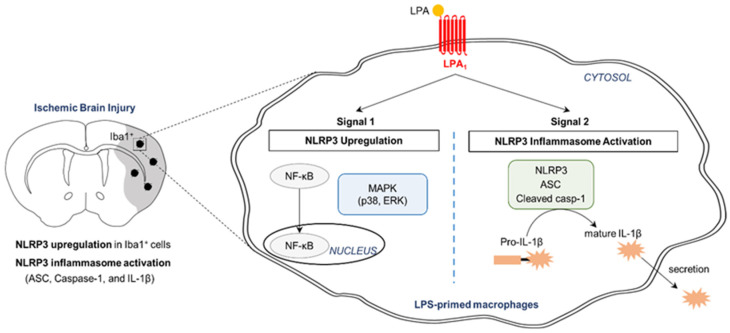
Schematic diagram showing the mechanism of NLRP3 inflammasome activation by LPA_1_. In injured brains after tMCAO challenge, NLRP3 is upregulated in the penumbra and ischemic core regions, particularly in cells expressing Iba1 (a marker of macrophages). NLRP3 inflammasome activation including ASC speck formation, caspase-1 activation, and IL-1β maturation occurs in injured brains. LPA_1_ contributes to NLRP3 upregulation (Signal 1) and NLRP3 inflammasome activation (Signal 2) in injured brains. In cultured macrophages, LPA_1_ also regulates NLRP3 upregulation and NLRP3 inflammasome activation (caspase-1 activation, IL-1β maturation, and IL-1β secretion) in LPS-primed macrophages, followed by LPA exposure. As underlying mechanisms, LPA_1_ regulates translocation of NF-κB into the nucleus and activation of p38 and ERK1/2 in these stimulated macrophages. It has been reported that LPA_1_ can regulate such underlying mechanisms in injured brains after ischemic challenge [4].

LPA_1_ is of great interest because it is being therapeutically pursued through ongoing clinical trials (Clinicaltrials.gov ID: NCT01766817; Clinicaltrials.gov ID: NCT02763969) for drug development to treat pulmonary fibrosis and psoriasis [33,34,35]. Other than these diseases, targeting LPA_1_ has been indicated to be beneficial for various diseases, including neuropathic pain and cerebral ischemia [4,6,36]. In particular, LPA_1_ antagonism can result in neuroprotection against an acute brain injury following transient ischemic challenge by modulating immune responses in the injured brain [4]. The current study addressed how LPA_1_ could contribute to an acute brain injury following ischemic challenge along with underlying molecular mechanisms (Figure 9). The pathogenic role of LPA_1_ in cerebral ischemia was associated with NLRP3 inflammasome activation, including NLRP3 upregulation, ASC speck formation, caspase-1 activation, and IL-1β maturation in a post-ischemic brain. This contribution of NLRP3 inflammasome activation to LPA_1_-dependent ischemic injury could be supported by the pathogenic role of NLRP3 inflammasome activation in a post-ischemic brain [37,38]. Either a genetic deficiency [37] or a pharmacological inhibition [38] might attenuate brain damages after ischemic challenge. As underlying molecular mechanisms, LPA_1_ signaling was found to be able to regulate NLRP3 inflammasome activation by activating NF-κB, ERK1/2, and p38 based on in vitro studies using cultured macrophages.

NLRP3 inflammasome activation can be regulated by priming signal (signal 1) and activation signal (signal 2) in macrophages [39]. Signal 1 is responsible for the upregulation of NLRP3 and IL-1β via NF-κB activation. Signal 2 is responsible for an assembly of NLRP3 inflammasome complex and caspase-1-dependent IL-1β maturation. A variety of endogenous/exogenous molecules can regulate NLRP3 inflammasome activation [40,41]. Recently, it was demonstrated that LPA signaling can regulate NLRP3 inflammasome activation in primed macrophages [18]. LPA can induce both NLRP3 upregulation and NLRP3 inflammasome activation in LPS-primed BMDMs, but not in unprimed BMDMs [18]. Moreover, LPA_5_, one of LPA receptors, can regulate both NLRP3 upregulation and NLRP3 inflammasome activation in LPS-primed cells, followed by LPA exposure [18]. The current in vitro study provided evidence that LPA_1_ could also regulate these events in LPS-primed macrophages, followed by LPA exposure. Importantly, the pathogenic role of LPA_1_ signaling in cerebral ischemia [4] could be closely associated with NLRP3 inflammasome activation (the current study). LPA amounts are increased in plasma samples of ischemic stroke patients [19] and injured brains of animal models following ischemic challenge [20], indicating that LPA may contribute to ischemic brain injury. Indeed, exogenous LPA can increase cortical infarction in rats after tMCAO challenge [42]. Suppressing LPA production with an autotaxin inhibitor can reduce brain infarction and neural cell apoptosis in rats with the same challenge [20]. It is of note that LPA can induce NLRP3 inflammasome activation as shown in our previous study [18] and the current study. It is also of note that both LPA_1_ [5,6] and NLRP3 inflammasome activation [16,17,37,38] can contribute to an ischemic brain injury. More importantly, the current in vivo study demonstrated that suppressing LPA_1_ activity with its specific antagonist attenuated NLRP3 upregulation and NLRP3 inflammasome activation in an injured brain following tMCAO challenge. Therefore, NLRP3 inflammasome activation could be an underlying mechanism of LPA_1_-mediated brain damage following ischemic challenge. The current study also indicated that LPA_1_ could be a novel regulator of NLRP3 inflammasome activation.

NF-κB, ERK1/2, and p38 MAPK seemed to be involved in the LPA/LPA_1_ signaling axis-dependent activation of macrophage NLRP3 inflammasome activation. All these signaling pathways can influence NLRP3 inflammasome activation as players for the priming signal to induce NLRP3 upregulation [26,27,29,30,31]. They are also well-known as effector pathways after LPA_1_ activation [32]. Indeed, the current in vitro study clearly showed that suppressing LPA_1_ activity by AM152 treatment attenuated the activation of NF-κB, ERK1/2, and p38 in LPS-primed BMDMs following LPA exposure. Interestingly, LPA_1_ can regulate the activation of all these signaling molecules in a post-ischemic brain [4]. Suppressing LPA_1_ activity with AM095 administration can attenuate NF-κB activation in the ischemic core region following tMCAO-challenge [4]. It can also attenuate the activation of ERK1/2 and p38 in an injured brain after tMCAO challenge [4]. Considering the influence of LPA_1_ signaling on these three effectors in BMDMs in vitro (the current study) and in post-ischemic brains in vivo [4], the role of LPA_1_ in NLRP3 inflammasome activation in post-ischemic brain observed in the current study might also be regulated by NF-κB, ERK1/2, and p38 signaling pathways.

In the current study, we revealed that LPA_1_ could regulate neuroinflammatory responses in post-ischemic brains through upregulating NLRP3 expression and promoting NLRP3 inflammasome activation. Such roles of LPA_1_ were reaffirmed in LPS-primed macrophages, followed by LPA exposure. In fact, neuroinflammation is a key pathogenic event in post-ischemic brains [43,44], and targeting neuroinflammation could be an appealing strategy for developing therapeutics to treat cerebral ischemia [45,46]. In this view, targeting NLRP3 inflammasome activation with LPA_1_ antagonists could be of interest. However, the multiphasic roles of inflammatory cells, such as neuroprotective and neuroharmful roles, should be considered because the wrong treatment at the wrong time could lead to detrimental effects [47,48]. During the acute phase, inflammatory cells, such as brain resident microglia and infiltrated macrophages, are mainly involved in neuronal damage by accelerating inflammatory cascades through the profound release of proinflammatory cytokines (i.e., tumor necrosis factor-α (TNF-α), IL-1β, and interleukin 6 (IL-6)) [47]. In contrast, at the later stage, these cells are also responsible for ischemic recovery [47]. Therefore, anti-inflammatory treatments might be mostly effective during the acute phase of ischemic challenge. Our recent study revealed that suppressing LPA_1_ activity could efficiently attenuate inflammatory responses in post-ischemic brains [4]. AM095, an LPA_1_ antagonist, attenuated activation of microglia and astrocytes, their proliferation, microglial NF-κB activation, and production of proinflammatory cytokines, such as TNF-α, IL-6, and IL-1β, in injured brains during the acute phase after tMCAO challenge (at 1 or 3 days after the challenge) [4]. These findings suggest that targeting LPA_1_ can lead to significant anti-inflammatory responses during the acute phase after ischemic challenge possibly through suppressing glial activation. However, whether LPA_1_ can be involved in the ischemic recovery during the later phase after ischemic challenge remains unclear.

In conclusion, the current study demonstrates NLRP3 inflammasome activation is an underlying mechanism for LPA_1_-mediated brain injury following ischemic challenge with experimental evidence for possible signaling pathways in this event. Other than cerebral ischemia, both NLRP3 inflammation and LPA_1_ signaling have been independently suggested as promising targets to develop therapeutics in various diseases, including tissue fibrosis and psoriasis. In this context, findings of the current study might be applied to understand how LPA_1_ can contribute to tissue injuries in these diseases.

## 4. Materials and Methods

### 4.1. Animals

Male ICR mice (six weeks old) were purchased from Orient Bio (Seongnam-Si, Gyeonggi-do, Korea). All animal experiments were approved by Lee Gil Ya Cancer and Diabetes Institute (LCDI) at Gachon University (animal protocol approval No.: LCDI-2019-0027, 1 March 2019) and performed in accordance with the Institutional Animal Care and Use guidelines. Mice were housed under controlled conditions: 12 h/12 h light/dark cycle, temperature of 22 ± 2 °C, and humidity of 60 ± 10%. They had free access to food and water through the experiment.

### 4.2. Transient Focal Cerebral Ischemia Challenge and AM095 Administration

Transient focal cerebral ischemia in mice was induced by MCAO for 90 min and reperfusion (‘tMCAO’) as described previously [49]. Briefly, mice were anesthetized with isoflurane (3% for induction and 1.5% for maintenance) with an air mixture of oxygen:nitrous oxide at ratio of 30:70%. Vertical neck incision was made and the right common carotid artery (CCA) was carefully separated from the vagus nerve. The external carotid artery and internal carotid artery were then carefully separated and MCAO was induced by inserting a silicon coated 5-0 monofilament (9-mm-long) toward the MCA through the internal carotid artery from CCA bifurcation. Mice were allowed to recover and subjected to an examination of intra-ischemic scores [50,51] to confirm successful MCAO at 1 h after occlusion. Monofilament was withdrawn at 90 min after occlusion to restore the blood flow under an anesthetic condition. Body temperature was controlled at 37 °C during surgery. To mitigate the post-operative pain, 2% lidocaine cream was applied to the surgical sites. To replenish a fluid loss during the surgery, physiological saline (10 mL/kg, i.p.) was administered after the surgery. For the sham-operated group, animals underwent the same anesthetic and surgical procedures without MCAO.

AM095 was used as an LPA_1_ antagonist for in vivo experiments. After MCAO surgery, mice were randomly divided into a vehicle (1% dimethyl sulfoxide in 10% Tween-80)-administered group and an AM095-administered group. Vehicle or AM095 (30 mg/kg) was administered into mice by oral gavage using a stomach tube immediately after reperfusion. The dosage of AM095 was set based on previously reported in vivo studies, including ours [4,52,53,54]. The administration was done by an investigator blinded to treatment groups. In the current study, one mouse from a vehicle-administered group died before an experimental endpoint and this dead mouse was excluded from the study.

### 4.3. Determination of Functional Neurological Deficit Score

Functional neurological deficit score was analyzed at one day after tMCAO challenge in a ’blinded fashion using a modified neurological severity score (mNSS) grade to assess deficits of motor, sensory, reflex, and balance functions in mice as previously described [55]. The mNSS grade ranged from zero point for normal to 18 points for the maximal deficit. The determined neurological deficit score data for all mice that were used for qPCR analysis, histological assessment, and Western blot analysis are shown in Figure S1.

### 4.4. Quantitative Real-Time PCR (qPCR) Analysis

Total RNAs were isolated from ipsilateral brains at one day after ischemic challenge using RNAiso plus (Takara, Kusatsu, Japan). Total RNAs (1 µg) were used to synthesize cDNAs by reverse transcription using an All-in-One First-Strand cDNA Synthesis SuperMix (TransGen Biotech, Haidian, China). qPCR analysis was done using a StepOnePlus^TM^ qPCR system (Applied Biosystems, Foster City, CA, USA) with Power SYBR Green PCR master mix (Life Technologies, Carlsbad, CA, USA). Expression levels of NLRP3 mRNA were calculated using the 2^−ΔΔCT^ method. Mouse β-actin housekeeping gene was used as a reference. The following primers were used: β-actin forward, 5′-AGCCTTCCTTCTTGGGTATG-3′; β-actin reverse, 5′-CTTCTGCATCCTGTCAGCAA-3′; NLRP3 forward, 5′-TCG CCC AAG GAG GAA GAA GAA-3′; NLRP3 reverse, 5′-TGA GAA GAG ACC ACG GCA GAA-3′.

### 4.5. Immunohistochemistry for NLRP3 and Double Immunofluorescence for NLRP3/Iba1 or ASC/NLRP3 

Brain samples for histological assessment were collected at one day after ischemic challenge. Mice were anesthetized with a mixture of Zoletil 50^®^ (10 mg/kg, i.m., Virbac Laboratories, Carros, France) and Rompun^®^ (3 mg/kg, i.m., Bayer HealthCare LLC, Shawnee Mission, KS, USA), perfused transcardially with ice-cold phosphate-buffered saline, and fixed with paraformaldehyde (PFA). Brains were removed, post-fixed with 4% PFA overnight, immersed in 30% sucrose solution, embedded in Tissue-Tek Optimal Cutting Temperature compound, and frozen on dry ice. These frozen brain samples were cut into 20-µm coronal sections using a cryostat (RD-2230, Roundfin, Liaoning, China).

For NLRP3 immunohistochemistry, brain sections were post-fixed in PFA, exposed to 0.01M sodium citrate at 90–100 °C and 1% H_2_O_2_, blocked with 1% fetal bovine serum (FBS), and incubated with a mouse anti-NLRP3 primary antibody (1:200, AdipoGen Life Sciences, San Diego, CA, USA) overnight at 4 °C, followed by labeling with a biotinylated secondary antibody (1:200, Santa Cruz Biotechnology, Dallas, TX, USA), for 2 h at room temperature (RT). These sections were further incubated with ABC reagent (1:100, Vector Laboratories, Burlingame, CA, USA). Signals were developed with a DAB kit (Dako, Santa Clara, CA, USA), rinsed with water, dehydrated with alcohol and xylene, and mounted with an Entellan media (Merck, Darmstadt, Germany).

For NLRP3/Iba1 double immunofluorescence, brain sections after blocking with 1% FBS were incubated with mouse anti-NLRP3 (1:100) and rabbit anti-Iba1 (1:500, Wako Pure Chemicals, Osaka, Japan) primary antibodies overnight at 4 °C. In case of ASC/NLRP3 double immunofluorescence, rabbit anti-ASC (1:200, AdipoGen Life Sciences) and mouse anti-NLRP3 (1:100) primary antibodies were used. Sections were then labeled with AF488- and Cy3-conjugated secondary antibodies (1:1000, Jackson ImmunoResearch West Grove, PA, USA) for 2 h at RT, followed by counterstaining with 4’,6-diamidino-2-phenylindole (DAPI) (Carl Roth, Karlsruhe, Germany). Labeled sections were mounted with VECTASHIELD^®^ (Vector Laboratories).

Bright-field or fluorescence images were photographed with a microscope equipped with a DP72 camera (BX53T, Olympus, Tokyo, Japan) or with a confocal microscope (Eclipse A1 Plus, Nikon, Tokyo, Japan). All representative images were prepared using Adobe Photoshop Elements 8. For quantification, three different photos (600 µm × 600 µm) of each brain region were taken in a blind fashion. The number of immunopositive cells in each photo was manually counted and then converted to the number of immunopositive cells per unit area (mm^2^). The mean was used for the number of immunopositive cells for the region of a single mouse. In case of ASC/NLRP3 double immunofluorescence, the number of ASC/NLRP3-double immunopositive cells of each photo (200 µm × 200 µm) was manually counted and then used for calculating % of cells with ASC/NLRP3 specks versus total cells (DAPI-positive cells).

### 4.6. BMDMs Primary Culture, Treatment, and LPA_1_ Knockdown

Bone marrow cells isolated from leg bones of ICR mice (male, 8 weeks old, Orient Co. Ltd.) were differentiated into BMDMs for three days by incubation with minimum essential medium alpha (α-MEM) containing recombinant mouse macrophage colony stimulating factor (30 ng/mL, R&D systems, Minneapolis, MN, USA) as described previously [56]. For experiments, BMDMs (5 × 10^6^ cells/well in a 6-well plate) were serum starved overnight by incubation with serum-free α-MEM. Cells were then primed with LPS (500 ng/mL, Sigma-Aldrich, St. Louis, MO, USA) for 4 h, followed by an exposure to LPA (1 µM, Avanti Polar Lipids, Birmingham, AL, USA) for 1 h. Fatty acid-free bovine serum albumin (0.1%, Sigma-Aldrich) was used as a vehicle.

To suppress LPA_1_ activity in vitro, BMDMs were serum starved overnight and treated with AM152 (another specific antagonist for LPA_1_) for 30 min. Cells were then primed with LPS and exposed to LPA. Alternatively, BMDMs were subjected to a transient transfection with LPA_1_ siRNA (Dharmacon, Lafayette, CO, USA) or control siRNA (Dharmacon) with Lipofectamine^®^ RNAiMAX (Life Technologies) under a serum-free and antibiotics-free condition, as previously described [18]. For experiments, transfected cells were serum starved, primed with LPS, and exposed to LPA.

### 4.7. Western Blot Analysis

Proteins were extracted from either the ipsilateral brain hemisphere at one day after ischemic challenge or cultured BMDMs using a neuronal protein extraction reagent (Thermo Fisher Scientific, Waltham, MA, USA). Protein samples were separated by SDS-PAGE and transferred to polyvinylidene difluoride (PVDF) membranes (Merck Millipore, Burlington, MA, USA). These membranes were blocked with 5% skim milk, incubated overnight with primary antibodies against NLRP3 (1:1000), pro-caspase 1 (1:1000, Abcam, Cambridge, UK), cleaved caspase-1 (1:1000, AdipoGen Life Sciences), pro-IL-1β (1:1000, Cell Signaling Technology, Danvers, MA, USA), mature IL-1β (1:1000, Abcam), phospho-NF-κB p65 (1:1000, Cell Signaling Technology), and β-actin (1:10000, Bethyl Laboratories, Montgomery, TX, USA). They were then incubated with horseradish peroxidase (HRP)-conjugated secondary antibodies (1:10000, Santa Cruz Biotechnology). Protein bands were detected using an enhanced chemiluminescence detection kit (Donginbiotech Co., Seoul, South Korea). Expression levels of target protein bands were quantified using ImageJ software (National Institute of Mental Health, Bethesda, MD, USA).

### 4.8. Measurement of IL-1β in Conditioned Medium

Cell-free supernatants were collected from treated cells by centrifugation at 1500 rpm for 5 min. They were then concentrated using a VIVASPIN 500 (Sartorius, Goettingen, Germany). Protein levels of secreted IL-1β into the culture medium were determined using IL-1β enzyme-linked immunosorbent assay kits (R&D systems, Minneapolis, MN, USA), according to the manufacturer’s instructions.

### 4.9. Determination of NF-κB Translocation

Cytosolic and nuclear proteins were extracted from BMDMs using ProteoExtract^®^ Subcellular Proteome Extraction Kit (Merck) according to the manufacturer’s instructions. Briefly, cells on a culture dish (60 mm^2^) were washed with Wash Buffer three times and incubated with ice-cold Extraction Buffer I containing protease inhibitor cocktail for 10 min at 4 °C under gentle agitation. Supernatant (fraction 1: cytosolic protein) was carefully removed and used for Western blot analysis. Extraction Buffer II containing protease inhibitor cocktail was added to the dish that was further incubated for 30 min at 4 °C under gentle agitation. Supernatant (fraction 2) that contain membrane/organelle protein was carefully removed. Finally, Extraction Buffer III containing protease inhibitor cocktail and Benzonase^®^Nuclease was added to the dish that was further incubated for 10 min at 4 °C under gentle agitation. Supernatant (fraction 3: nuclear protein) was carefully removed and used for Western blot analysis. Cytosolic and nuclear proteins were subjected to SDS-PAGE gel electrophoresis, transferred into PVDF membranes, and blocked. These membranes were incubated with primary antibodies against NF-κB p65 (1:1000, Cell Signaling Technology), β-actin (1:10000), and histone H3 (1:1000, Abcam) and incubated with HRP-conjugated secondary antibodies (1:10000).

### 4.10. Statistical Analysis

All data analyses were performed using GraphPad Prism 7 (GraphPad Software Inc., La Jolla, CA, USA). Date are presented as mean ± S.E.M. Statistical significance was analyzed by either Student′s *t*-test between two groups or one-way ANOVA, followed by a Newman–Keuls post hoc test, for comparisons among groups. Statistical significance was considered when *p*-value was less than 0.05.

## Supplementary Materials

The following are available online at https://www.mdpi.com/1422-0067/21/22/8595/s1, Figure S1: Administration of a lysophosphatidic acid receptor 1 (LPA_1_) antagonist, AM095, reduced neurological deficit score in mice after transient middle cerebral artery occlusion (tMCAO) challenge. Figure S2: AM152, an LPA_1_ antagonist, does not inhibit NF-κB phosphorylation in lipopolysaccharide (LPS)-primed BMDMs, followed by lysophosphatidic acid (LPA) exposure.

## Abbreviations

ASC	Apoptosis-associated speck-like protein containing a caspase recruitment domain
BMDMs	Bone marrow-derived macrophages
CCA	Common carotid artery
DAPI	4′,6-Diamidino-2-phenylindole
ELISA	Enzyme-linked immunosorbent assay
ERK1/2	Extracellular signal-regulated kinase 1/2
HRP	Horseradish peroxidase
Iba1	Ionized calcium-binding adapter molecule 1
IL-1β	Interleukin 1β
IL-6	Interleukin 6
JNK	c-Jun N-terminal kinase
LPA_1_	Lysophosphatidic acid receptor 1
LPS	Lipopolysaccharide
MAPK	Mitogen-activated protein kinase
α-MEM	Minimum Essential Medium alpha
mNSS	Modified neurological severity score
NF-κB	Nuclear factor-κB
NLRP3	Nucleotide-binding oligomerization domain-like receptor family pyrin domain containing 3
PFA	Paraformaldehyde
PVDF	Polyvinylidene difluoride
siRNA	Small interfering RNA
tMCAO	Transient middle cerebral artery occlusion
TNF-α	Tumor necrosis factor-α
