# Supplementary material for: NLRP3 Inflammasome Activation Is Involved in LPA1-Mediated Brain Injury after Transient Focal Cerebral Ischemia
Source: Int J Mol Sci. 2020 Nov 14;21(22):8595. doi: 10.3390/ijms21228595 (PMC7697439; doi:10.3390/ijms21228595)
Supplement: Supplementary file 1 [file ijms-21-08595-s001.pdf]

Supplementary information:

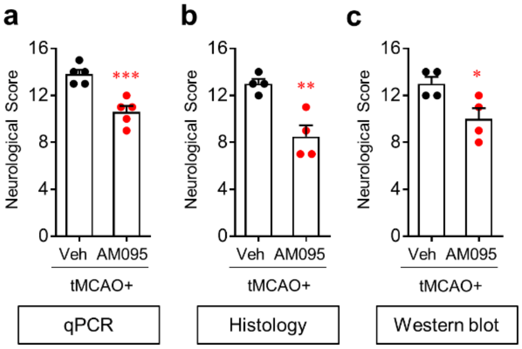

Figure S1. Administration of a lysophosphatidic acid receptor 1 (LPA_1) antagonist, AM095, reduced neurological deficit score in mice after transient middle cerebral artery occlusion (tMCAO) challenge. Mice were challenged with tMCAO. AM095 (30 mg/kg, p.o.) was administered into mice immediately after reperfusion. Neurological deficit score ('Neurological Score') was analyzed at 1 day after tMCAO challenge using a modified neurological severity score (mNSS) grade. Neurological deficit scores of mice used for quantitative Real-Time PCR (qPCR) (**a**), histological (**b**), and Western blot (**c**) analyses in the current study. $^{*}p < 0.05$, $^{**}p < 0.01$, and $^{***}p < 0.001$ versus vehicle-administered tMCAO group.

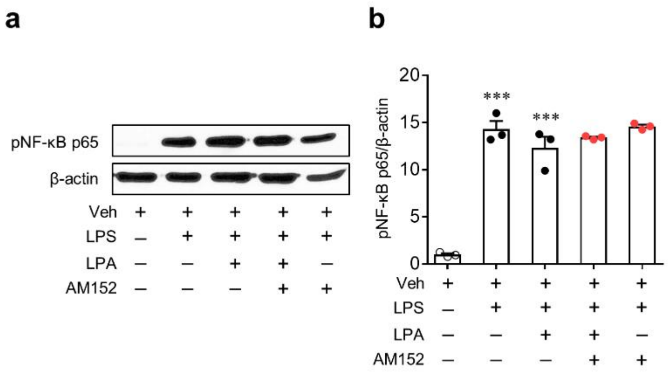

Figure S2. AM152, an LPA_1 antagonist, does not inhibit NF-κB phosphorylation in lipopolysaccharide (LPS)-primed BMDMs followed by lysophosphatidic acid (LPA) exposure. Cells were primed with LPS (500 ng/mL) for 4 h followed by an exposure to LPA (1 μM) for 1 h. NF-κB phosphorylation was determined by Western blot analysis using an antibody against phosphorylated NF-κB p65 (pNF-κB p65). Representative Western blots of pNF-κB p65 and β-actin (**a**) and quantification of NF-κB phosphorylation (**b**) are shown. n = 3 per group. $^{***}p < 0.001$ versus control BMDMs (Veh).
